# Unexplained health inequality – is it unfair?

**DOI:** 10.1186/s12939-015-0138-2

**Published:** 2015-01-31

**Authors:** Yukiko Asada, Jeremiah Hurley, Ole Frithjof Norheim, Mira Johri

**Affiliations:** Department of Community Health and Epidemiology, Dalhousie University, 5790 University Avenue, Halifax, Nova Scotia B3H1V7 Canada; Department of Economics and Centre for Health Economics and Policy Analysis, McMaster University, Hamilton, Ontario L8S4M4 Canada; Department of Research and Development, Haukeland University Hospital, Jonas Liesvei 65, 5021 Bergen, Norway; Centre de Recherche du Centre Hospitalier de l’Université de Montréal (CRCHUM), Tour Saint-Antoine, Porte S03-458, 850, rue St-Denis, Montreal, Quebec H2X0A9 Canada; Département d’administration de la santé, Université de Montréal, C.P. 6128, succursale Centre-ville, Montreal, Quebec H3C3J7 Canada

**Keywords:** Health inequalities, Health disparities, Health inequities, Measurement, Ethics

## Abstract

**Introduction:**

Accurate measurement of health inequities is indispensable to track progress or to identify needs for health equity policy interventions. A key empirical task is to measure the extent to which observed inequality in health – a difference in health – is inequitable. Empirically operationalizing definitions of health inequity has generated an important question not considered in the conceptual literature on health inequity. Empirical analysis can explain only a portion of observed health inequality. This paper demonstrates that the treatment of unexplained inequality is not only a methodological but ethical question and that the answer to the ethical question – whether unexplained health inequality is unfair – determines the appropriate standardization method for health inequity analysis and can lead to potentially divergent estimates of health inequity.

**Methods:**

We use the American sample of the 2002–03 Joint Canada/United States Survey of Health and measure health by the Health Utilities Index (HUI). We model variation in the observed HUI by demographic, socioeconomic, health behaviour, and health care variables using Ordinary Least Squares. We estimate unfair HUI by standardizing fairness, removing the fair component from the observed HUI. We consider health inequality due to factors amenable to policy intervention as unfair. We contrast estimates of inequity using two fairness-standardization methods: direct (considering unexplained inequality as ethically acceptable) and indirect (considering unexplained inequality as unfair). We use the Gini coefficient to quantify inequity.

**Results:**

Our analysis shows that about 75% of the variation in the observed HUI is unexplained by the model. The direct standardization results in a smaller inequity estimate (about 60% of health inequality is inequitable) than the indirect standardization (almost all inequality is inequitable).

**Conclusions:**

The choice of the fairness-standardization method is ethical and influences the empirical health inequity results considerably. More debate and analysis is necessary regarding which treatment of the unexplained inequality has the stronger foundation in equity considerations.

**Electronic supplementary material:**

The online version of this article (doi:10.1186/s12939-015-0138-2) contains supplementary material, which is available to authorized users.

## Introduction

Inequalities and inequities in health care and health outcomes continue to be in the center stage of health policy in many jurisdictions. Accurate measurement of inequalities and inequities is indispensable to track progress or to identify needs for policy interventions [1,2]. Regular reporting of health inequalities and inequities requires ongoing data and methodological improvement. Measurement of health inequities is more challenging than that of health inequalities not only for their requirements for data on determinants of health [3] but also for ethical considerations. Health inequities are a subset of ethically problematic health inequalities – differences in health –, and their measurement demands a definition of health inequity and operationalization of the chosen definition in the measurement exercises [4,5].

To date, no single, agreed-up definition of health inequities exists. Alternative definitions of health inequity can be distinguished by the sources of health inequality each classified as ethically acceptable and unacceptable. For example, Braveman and Gruskin define health equity as “the absence of systematic disparities in health … between social groups who have different levels of underlying social advantage/disadvantage” ([6], p. 254). This view thus regards inequalities associated with social advantage as ethically unacceptable. In contrast, equal opportunity for health, a definition gaining popularity in the health economics literature [7-10], considers health inequality due to factors beyond individual control is unfair. In this view, factors within individual control are ethically acceptable sources of inequality.

Given that the ultimate focus of policy concern is health inequities, a key empirical task is to measure the extent to which observed inequality in health is inequitable. This requires an integration of the conceptual and empirical literatures on health inequity [4,5,11,12]. Empirically operationalizing definitions of health inequity has generated an important question not considered in the conceptual literature on health inequity noted above [7,11,12]. Empirical analysis can explain only a portion of observed health inequality. The presence of large unexplained variation in health regression models is no news to methodologists, who typically consider it as a data or methodological limitation. However, in the measurement of health inequity, a question arises as to how we should classify unexplained health inequality – fair or unfair. This ethical question is unavoidable in such empirical exercises, and different answers to this question can result in divergent health inequity results, some of which are fundamental to health equity policy, such as how much health inequity exists in the population, and to what degree observed health inequality is inequitable. Despite potentially large policy implications, the issue of unexplained health inequality has not received sufficient attention in health services and population health research and policy.

The goal of this paper is to demonstrate that the answer to the ethical question – whether unexplained health inequality is unfair – determines the choice of the standardization method and can lead to potentially divergent estimates of health inequity. In the next section, we explain how this question arises in the assessment of health inequities and articulate how answers to this question lead to particular methodological choices. We then demonstrate the importance of this ethical question empirically using the Joint Canada/United States Survey of Health (JCUSH) [13], which is typical of the data available for health inequity analysis. Our analysis shows that different ethical judgments regarding unexplained health inequality lead to substantial differences in estimates of health inequity. We conclude by discussing future research directions to enhance understanding of this issue.

### Ethical judgments regarding unexplained health inequality in health inequity analysis

The issue of unexplained health inequality arises in an effort to be transparent and explicit about the definition of health inequity when empirically measuring health inequity. Measuring health inequity requires individual-level data to model variation in health at the individual level. Assuming that we have such individual-level health survey data, we begin by quantifying the amount of inequality in the distribution of observed health across individuals. We can use a univariate inequality index (e.g., Gini index). This provides a measure of the total amount of health inequality in the population.

To measure health inequity, we must quantify the distribution of *unfair* health across individuals in the population, that is, unfair health inequality. Unfair health, however, is not directly observable. To estimate it from observed health, we first model variation in health. The goal is to statistically explain as much variation in health as possible with the data at hand. This enables us to partition variation in health into that attributable to factors considered fair, or legitimate, sources of variation, and that attributable to factors considered unfair, or non-legitimate, source of variation. In other words, to define health inequities we need to look at causes of health inequalities [11].

As an example, let us consider a popular definition of health inequity, policy amenability, which argues that health inequality due to factors amenable to policy intervention is unfair [14]. We classify each variable in our data as a legitimate (ethically acceptable) source of inequality – that is, it is not amenable to policy intervention – or an illegitimate (ethically unacceptable) source of inequality – that is, it is amenable to policy intervention. Table 1 is an example of such legitimate-illegitimate classification based on the perspective of policy amenability. We assume age largely represents the biological association with health and treat it as the only variable that is not amenable to policy intervention, and thus, a legitimate source of variation in health. We classify all other variables as amenable to policy because: (a) it is possible to change the distribution of the variable (e.g., education, income), or (b) even when it is not possible to change the distribution of the variable, it is in principle possible to change how society treats people with the characteristic (e.g., for race and sex, it is possible to eliminate racial or sex discrimination). Age and sex capture both biology and social policy, and the asymmetrical treatment stems from our assumption as to which effect each of these variables represents most.

**Table 1:** **Legitimate-illegitimate classification of variables according to the perspective of policy amenability**

**Variable**	**Legitimate vs. illegitimate classification**
Demographics status	
Age	Legitimate
Sex	Illegitimate
Marital status	Illegitimate
Race	Illegitimate
Country of birth	Illegitimate
Health behaviour	
Smoker type and history	Illegitimate
BMI	Illegitimate
Frequency of physical activity	Illegitimate
Socioeconomic status	
Household income	Illegitimate
Education	Illegitimate
Health care factors	
Has regular medical doctor	Illegitimate
Unmet need	Illegitimate
High blood pressure management	Illegitimate
Asthma medication management	Illegitimate
Pharmaceutical insurance	Illegitimate
Health insurance type	Illegitimate

BMI: body mass index.

Variables are those we include in our analysis using the Joint Canada/United States Survey of Health (JCUSH).

“Policy amenability” argues that health inequality due to factors amenable to policy intervention is unfair [14].

A legitimate source of health inequality means that the variable is not amenable to policy, thus, resulting health inequality is ethically acceptable.

An illegitimate source of health inequality means that the variable is amenable to policy, thus, resulting health inequality is ethically unacceptable.

Classifications like that in Table 1 generate intense debate for at least two reasons. First, defining health inequity in this way assumes causality between health and the other variables, which cannot always be established empirically due to data and methodological limitations. Second, people debate passionately whether a particular source is legitimate or illegitimate. Our particular choices presented in Table 1 are only for illustrative purposes. The key point here is that, to estimate unfair health, one needs to classify variables as legitimate or illegitimate according to a chosen definition of health inequity.

Having classified each variable, we then remove the influence of the fair component – legitimate variables according to a chosen definition of health inequity – on the observed health through *fairness-standardization.* This leaves us with only inequality due to unfair sources. Fairness-standardization is similar to age-standardization in epidemiological studies, which removes the influence of age when estimating mortality rates, but in this context, standardization removes the influence of all legitimate sources of inequality. Consequently, the standardization generates the inequitable distribution of health in the population. The amount of inequity is then quantified by applying the same inequality index as above to this distribution of unfair health. Despite the use of the same mathematical index, the measure here is an index of inequity, as opposed to simply inequality, as it quantifies the distribution of unfair health.

For fairness-standardization, two methods are available: direct and indirect. As we show below, the choice of the standardization method is closely connected to ethical judgments regarding unexplained inequality.

Both direct and indirect standardization methods are based on the notion that the observed health consists of legitimate, illegitimate, and unexplained components:

$$ Observed\ HUI= Legitimate+ Illegitimate+ Unexplained $$

Using the direct standardization method, we predict unfair health directly by allowing only the illegitimate variables alone to influence the predictions. To do so, we purge the influence of legitimate variables by setting the value of these variables constant (expressed with the bar in the equation) during the prediction and ignore the unexplained component:

$$ Unfa{\widehat{ir\ HUI}}_{direct}=\overline{Legitimate}+ Illegitimate $$

As is clear, this produces a distribution in which the only source of variation in predicted levels of health arises from variation across individuals in illegitimate factors.

Using the indirect standardization, we first predict fair health by allowing only the legitimate variables to influence the predictions. To do so, we purge the influence of illegitimate variables (by holding their values at a constant during prediction) and ignore the unexplained component:

$$ F\widehat{air\ H}UI= Legitimate+\overline{Illegitimate} $$

We then calculate unfair health by subtracting the estimate of fair health from the observed health and adding the mean health of the population:

$$ \begin{array}{l} Unfai{\widehat{r\ HUI}}_{indirect}\\ {}= Observed\ HUI-F\widehat{air\ H}UI+ Populatio{n}^{\hbox{'}}s\  mean\ HUI\\ {}=\left( Legitimate+ Illegitimate+ Unexplained\right)- Legitimate + Populatio{n}^{\hbox{'}}s\  mean\ HUI\\ {}= Illegitimate+ Unexplained+ Populatio{n}^{\hbox{'}}s\  mean\ HUI\end{array} $$

This addition of the mean health of the population is conventional [15] and ensures that the distributions of the observed health and the unfair health have the same mean value.

For both standardization methods, we can choose any values at which to hold the relevant variables constant (legitimate variables for direct standardization and illegitimate for indirect standardization). But the choice reflects an ethical and policy judgment regarding the reference attributes by which we assess health inequity. For example, for the definition of policy amenability discussed above, we can hold each relevant variable at the category to which policies might reasonably aim (e.g., education at “high school”), or we could set the level to the healthiest category in the population (e.g., education at “university or college certificate”). Whether we should assess health inequity against a modest or ambitious goal depends on which differences in health we consider as unfair and what reference we consider as an appropriate policy goal. Just as the legitimate-illegitimate classification of variables, the choice of reference values can generate debate. For the purpose of this paper, which compares the two standardization methods, we set reference values equal to the modest goals as an example.

Importantly for the focus of this paper, notice that the unfair health estimated by the direct standardization does not include the unexplained component while the unfair health estimated by the indirect standardization does. The larger the unexplained component is, the greater the discrepancy is between unfair health estimated by these two standardization methods.

Notice further that the choice of the standardization methods implies ethical judgments: using the direct standardization, we regard unexplained variation in inequality as ethically acceptable, and using the indirect standardization, we regard it as unfair. Although this issue has been raised in the health economics literature [7,11,12], there has been little appreciation for these ethical judgments in the public health and health policy literatures. Below we illustrate how much difference these standardization methods can make in estimates of health inequity using typically available survey data.

## Methods

### Data

We estimate health inequity using the 2002–03 Joint Canada/United States Survey of Health (JCUSH), a cross-sectional population health survey jointly conducted by Statistics Canada and the U.S. National Center for Health Statistics [13]. The JCUSH questionnaire included questions regarding health status, health care utilization, health behaviour, socioeconomic status, and health insurance status. The target population was non-institutionalized Canadian and U.S. household residents aged 18 and older. The JCUSH used a complex sampling design with stratification by geographic region and oversampling of respondents aged 65 and over.

For simplicity and the ease of exposition, in this paper we present the results for the American sample only. The analysis using the Canadian sample yielded the same key methodological findings (available from the authors upon request). The original American sample of the JCUSH is 5,183 (response rate: 50.2%). We exclude observations with missing values (typically less than 4% of observations), except income (19.8%), for which we create “income missing” category. We also exclude 48 observations with scores of the Health Utilities Index (HUI), our measure of health, less than or equal to zero. The final sample size for our analysis is 4,328.

### Variables

#### Health

We measure health by the Health Utilities Index Mark 3 (HUI), a well validated and widely used generic health-related quality of life measure [16]. The HUI measures the respondent’s functional levels in eight dimensions (vision, hearing, speech, mobility, dexterity, emotion, cognition, and pain) and converts his or her functional levels into a health-related quality-of-life score based on preferences of the general public (as opposed to the respondent’s preferences) over health states. One advantage of the HUI is that it is possible to identify when a difference in scores is meaningful for policy purposes. A difference of 0.030 or greater is meaningful or important [17], indicating the difference large enough to justify a recommendation for an intervention to achieve such an increment in health [18]. The observed distribution of HUI scores in the full sample range from −0.360 to 1.000 on a scale in which 0.000 represents being dead and 1.000 represents perfect health, and negative scores indicates health states worse than dead. For our analysis, we use only observations with zero or positive HUI scores as the Gini index, by which we measure univariate inequality and inequity, allows only non-negative values for the variable being analyzed [19].

### Attributes known to be associated with health

We use a number of attributes known to be associated with health and available from the JCUSH: demographic status, health behaviour, socioeconomic status, and health care system factors, including the availability of basic health care, quality of health care, and health care insurance. We tested for interactions among these variables and retained the interaction terms between smoking and income and between body mass index (BMI) and education, which remain statistically significant at the 5% level in the final model.

### Quantifying health inequality and inequity

We use the Gini coefficient to quantify health inequality and inequity [4,20]. The Gini coefficient takes values between zero (perfectly equal distribution) and one (most unequal). The Gini coefficient is widely used in the income inequality literature and has also been applied to quantify the distribution of health [21]. The Gini coefficient assumes that the underlying variable is measured at the ratio scale level. The HUI is an interval-scale measure, so our application of the Gini to the HUI violates this assumption. In practice, however, many inequality analyses apply the Gini to health measures that do not strictly satisfy this assumption, and given that the choice of inequality measure is not central to the main focus of our analysis, we believe our use of the Gini is reasonable. Although the 0–1 index of the Gini coefficient itself does not give an intuitive interpretation, twice the value of the Gini coefficient indicates the proportion of the expected mean difference between two randomly selected persons in the population [22]. For example, a value of 0.100 for the Gini coefficient with the mean HUI, 0.800, indicates that the expected difference in the HUI from two randomly drawn persons in this population is twice 0.100 (i.e., 0.200) of the mean HUI, 0.800 (i.e., 0.160). When the Gini coefficient in the population indicates the expected difference in the HUI from two randomly drawn persons equal to or greater than 0.030, the minimum magnitude for a difference in HUI scores to be policy relevant, we consider this inequality or inequity as policy relevant.

### Analysis

The analysis proceeds with the following three steps. First, we estimate the magnitude of inequality in the observed HUI across individuals using the Gini coefficient. Second, we model variation in the observed HUI. Third, based on the definition of policy amenability, as discussed above, and using the direct and indirect standardization methods, we estimate unfair HUI for each individual and quantify the magnitude of inequity using the Gini coefficient. In both standardization methods, we hold relevant variable at the category to which policies might reasonably aim (see Additional file 1).

Modeling the distribution of the HUI is challenging because the HUI is bounded (between 0.000 and 1.000), it spikes at 1.0 (in our JCUSH sample, about 25% of the observations have HUI=1), and it is left-skewed. Researchers have recommended a number of alternative statistical methods to empirically model the distribution of HUI, including Ordinary Least Squares (OLS), Tobit, censored least absolute deviation (CLAD), two-part models, and latent class models, with no consensus regarding the best approach [23-26]. In this paper we present results from the OLS because OLS performed well relative to two-part models and CLAD in our sensitivity analysis and is easier to understand than the alternatives^a^.

We weight all analyses using the sample weights provided by the JCUSH. To estimate variance accounting for the JCUSH’s complex survey design, we use the balanced repeated replication methods with balanced repeated replication weights provided by Statistics Canada and the US National Center for Health Statistics. We consider p<0.05 as statistically significant. We use Stata 11 for all analyses [27,28].

## Results

### Sample characteristics

Sample characteristics and the average HUI across subgroups mostly follow expected patterns (Table 2). The average HUI is lower among older age groups; those separated, divorced, or widowed; black or other racial group; those with unmet need; those without pharmaceutical insurance; and those with Medicaid only. The average HUI does not differ much by sex or country of birth. Those with healthy behaviours and high socioeconomic status, measured by income or education, have higher average HUI. Those with no regular medical doctor and no health insurance have higher average HUI than those with regular medical doctor and health insurance, which may indicate younger age and less demand for health care among this group. High demand for health care may be a factor for lower average HUI among those with high blood pressure or asthma and received treatment or medication in the last 12 months than those with such conditions but who did not obtain treatment or medication.

**Table 2:** **Sample characteristics**

	**N (%)**	**HUI**
Total sample	4, 328(100)	0.869
Demographics status		
Age (year)		
18-44	1,962(45.33)	0.910
45-64	1,470(33.96)	0.856
65+	896(20.70)	0.800
Sex		
Men	1,899(43.88)	0.881
Women	2,429(56.12)	0.860
Marital status		
Married or common law partner	2,443(56.45)	0.888
Separated, divorced, or widowed	1,094(25.28)	0.812
Single	791(18.28)	0.889
Race		
White	3,384(78.19)	0.874
Other	500(11.55)	0.842
Black	332(7.67)	0.843
Asian	112(2.59)	0.918
Country of birth		
Foreign born	614(14.19)	0.867
Native born	3,714(85.81)	0.869
Health behaviour		
Smoker type and history		
Never smoked	2,259(52.20)	0.889
Former smoker and started smoking at or after 18 years	717(16.57)	0.858
Former smoker and started smoking before 18 years	342(7.90)	0.817
BMI		
Underweight	96(2.22)	0.820
Normal weight	1,864(43.07)	0.890
Overweight	1,455(33.62)	0.880
Obese	913(21.10)	0.813
Frequency of physical activity		
Regular	2,518(58.18)	0.907
Occasional	736(17.01)	0.885
Infrequent	1,074(24.82)	0.768
Socioeconomic status		
Household income		
Lowest income quintile	665(15.37)	0.769
Lower middle income quintile	696(16.08)	0.855
Middle income quintile	620(14.33)	0.894
Higher middle income quintile	726(16.77)	0.909
Highest middle income quintile	763(17.63)	0.930
Income missing	858(19.82)	0.852
Education		
Less than high school	431(9.96)	0.756
High school graduate	1,569(36.25)	0.856
Non-university/college certificate	635(14.67)	0.867
University/college certificate	1,693(39.12)	0.911
Health care factors		
Has regular medical doctor		
No	786(18.16)	0.890
Yes	3,542(81.84)	0.864
Unmet need		
No	3,816(88.84)	0.885
Yes	512(11.83)	0.753
With high blood pressure and received treatment in the last 12 months		
No	54(1.25)	0.820
Yes	832(19.22)	0.788
No high blood pressure	3,442(79.53)	0.889
With asthma and received medication in the last 12 months		
No	190(4.39)	0.882
Yes	280(6.47)	0.784
No asthma	3,858(89.14)	0.875
Has pharmaceutical insurance		
No	881(20.36)	0.846
Yes	3,447(79.64)	0.875
Health insurance type (US only)		
No insurance	443(10.24)	0.851
Medicaid only	160(3.70)	0.677
Non-Medecaid public only including Medicare	254(5.87)	0.758
Private plus public including Medicare	818(18.90)	0.811
Private only	2,653(61.30)	0.912

Data source: Joint Canada/United States Survey of Health (JCUSH).

BMI: body mass index; HUI: Health Utilities Index.

BMI is based on the World Health Organization. Underweight: <18.5 kg/m^2^; normal weight: 18.5-24.9 kg/m^2^; overweight: 25-30 kg/m^2^; obese >30 kg/m^2^.

HUI estimates are weighted and unadjusted.

### Modeling variation in health (HUI)

The fit of our model is comparable to other work describing the variation in the HUI (adjusted R^2^: 0.258, Table 3) [29,30]. Among the demographic variables, only age is statistically significant. Lack of statistical significance of race is somewhat counter-intuitive but confirms other studies using the JCUSH (e.g., [30]). When we add socioeconomic variables to demographic variables, race becomes statistically insignificant, and, after introducing health care supply variables, the sign of the coefficient for black flips from negative to positive. All health behaviour variables (smoker type, BMI, and physical activity) and socioeconomic variables (income and education) show statistically significant effects on the HUI, either individually or through interactions. All health care supply variables are statistically significant, with the unmet need variable showing the largest coefficient (−0.110), followed by health insurance type (−0.092 for Medicaid only with no insurance as the reference).

**Table 3:** **Results of ordinary least squares regression model for the health utilities index**

	**Coefficient (95% CI)**	**p-value**
Age (years, reference: 18-44)		0.000
45-64	-0.044(-0.057, -0.030)	0.000
65+	-0.013(-0.041, 0.015)	0.362
Male	-0.001(-0.012, 0.010)	0.890
Marital status (reference: single)		0.064
Married or common law partner	-0.005(-0.010, 0.019)	0.554
Separated, divorced, or widowed	-0.014(-0.034, 0.005)	0.155
Race (reference: White)		0.342
Other	-0.006(-0.028, 0.016)	0.601
Black	-0.016(-0.005, 0.037)	0.127
Asian	-0.011(-0.046, 0.024)	0.546
Foreign born	-0.006(-0.013, 0.026)	0.515
Smoking (reference: never smoked)		0.059
Former smoker and started smoking at or after 18 years	-0.052(-0.107, 0.002)	0.060
Former smoker and started smoking before 18 years	-0.086(-0.170, 0.003)	0.043
Current smoker and started smoking at or after 18 years	-0.015(-0.066, 0.036)	0.558
Current smoker and started smoking before 18 years	-0.070(-0.140, 0.000)	0.050
BMI (reference: normal weight)		0.053
Underweight	-0.166(-0.283, 0.048)	0.006
Overweight	-0.025(-0.084, 0.034)	0.402
Obese	-0.086(-0.084, 0.040)	0.485
Frequency of physical activity (reference; regular)		0.000
Occasional	-0.012(-0.024, 0.001)	0.069
Infrequent	-0.083(-0.099, -0.066)	0.000
Household income (reference: lowest income quintile)		0.122
Lower middle income quintile	-0.021(-0.009, 0.050)	0.169
Middle income quintile	-0.038(-0.010, 0.067)	0.009
Higher middle income quintile	-0.036(-0.008, 0.064)	0.011
Highest middle income quintile	-0.037(-0.008, 0.065)	0.011
Income missing	-0.027(-0.001, 0.056)	0.056
Education (reference: less than high school)		0.026
High school graduate	-0.007(-0.035, 0.049)	0.737
Non-university/college certificate	-0.032(-0.013, 0.078)	0.162
University/college certificate	-0.029(-0.013, 0.070)	0.176
Has regular medical doctor	-0.021(-0.036,-0.006)	0.005
Presence of self-reported unmet need	-0.110(-0.133,-0.087)	0.000
Treatment for high blood pressure in the last 12 months (reference: no treatment)		0.000
Received treatment	-0.023(-0.081, 0.034)	0.424
No high blood pressure	-0.020(-0.034, 0.073)	0.475
Medication for asthma in the last 12 months (reference: no medication)		0.008
Received medication	-0.020(-0.076,-0.004)	0.031
No asthma	-0.000(-0.026, 0.026)	0.999
Has pharmaceutical insurance	-0.032(-0.051, 0.013)	0.001
Health insurance type (US only, reference: no insurance)		0.000
Medicaid only	-0.092(-0.146,-0.039)	0.001
Non-Medicaid public only including Medicare	-0.052(-0.092,-0.013)	0.010
Private plus public including Medicare	-0.038(-0.074, 0.001)	0.043
Private only	-0.035(-0.008, 0.062)	0.010
Smoking x household income (reference: never smoked x lowest income quintile)		0.024
Former smoker and started smoking at or after 18 years		
x Lower middle income quintile	0.011(-0.063, 0.085)	0.770
x Middle income quintile	0.038(-0.024, 0.101)	0.225
x Higher middle income quintile	0.050(-0.010, 0.111)	0.101
x Highest middle income quintile	0.062(0.000, 0.124)	0.050
x Income missing	0.086(-0.003, 0.122)	0.064
Former smoker and started smoking before 18 years		
x Lower middle income quintile	0.041(-0.059, 0.141)	0.418
x Middle income quintile	0.062(-0.035, 0.160)	0.210
x Higher middle income quintile	0.105(0.015, 0.194)	0.023
x Highest middle income quintile	0.094(0.004, 0.184)	0.041
x Income missing	-0.003(-0.110, 0.105)	0.962
Current smoker and started smoking at or after 18 years		
x Lower middle income quintile	0.027(-0.035, 0.088)	0.396
x Middle income quintile	-0.022(-0.085, 0.040)	0.487
x Higher middle income quintile	-0.010(-0.072, 0.053)	0.757
x Highest middle income quintile	0.025(-0.033, 0.084)	0.393
x Income missing	0.002(-0.061, 0.065)	0.955
Current smoker and started smoking before 18 years		
x Lower middle income quintile	0.063(-0.016, 0.142)	0.116
x Middle income quintile	0.002(-0.100, 0.103)	0.976
x Higher middle income quintile	0.027(-0.058, 0.112)	0.531
x Highest middle income quintile	0.094(-0.007, 0.180)	0.034
x Income missing	0.002(-0.041, 0.141)	0.282
BMI x education (reference: normal weight x less than high school)		0.005
Underweight		
x High school graduate	0.108(-0.038, 0.255)	0.147
x Non-university/college certificate	0.159(-0.021, 0.340)	0.083
x University/college certificate	0.176(-0.052, 0.301)	0.006
Overweight		
x High school graduate	0.051(-0.012, 0.113)	0.112
x Non-university/college certificate	0.014(-0.051, 0.080)	0.665
x University/college certificate	0.022(-0.040, 0.083)	0.490
Obese		
x High school graduate	-0.001(-0.068, 0.067)	0.985
x Non-university/college certificate	-0.051(-0.128, 0.025)	0.190
x University/college certificate	0.013(-0.051, 0.078)	0.683
Constant	0.919(0.839, 1.000)	0.000
Sample size	4328	
Adjusted R-squared	0.258	

Data source: Joint Canada/United States Survey of Health (JCUSH).

CI: confidence interval; BMI: body mass index.

P-value for each variable category is from t-test; p-value for the reference category is from F-test for all categories of each variable.

Analysis is weighted. Standard errors are adjusted for the complex survey design.

### Health inequality

**Figure 1:**
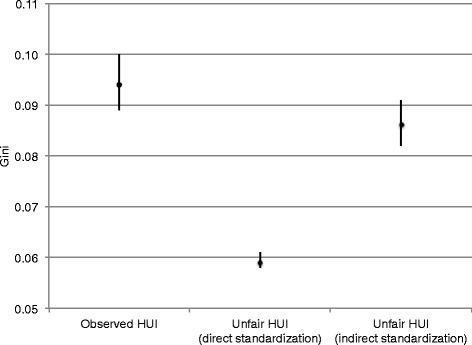
**Magnitude of health inequality and health inequity estimated by the direct and indirect fairness standardization.** Data source: Joint Canada/United States Survey of Health (JCUSH). Analysis is weighted. Standard errors are adjusted for the complex survey design. Gini coefficient takes values between zero (most equal) and one (most unequal). The use of the direct standardization implicitly regards unexplained variation in inequality as ethically acceptable, and the use of the indirect standardization implicitly regards it as unfair.

The far left data point of Figure 1 shows the magnitude of health inequality. The Gini coefficient for the distribution of the observed HUI is 0.094 (95% CI: 0.089, 0.100), and the mean HUI value for this distribution is 0.880 (95% confidence interval [CI]: 0.873, 0.886). Based on this information, the expected mean difference in the HUI of two randomly selected individuals is 0.165, which notably larger than the minimally policy significant difference in the HUI of 0.030.

### Health inequity – the direct vs. indirect fairness-standardization method

As shown in Figure 1, the choice of the standardization method makes a substantial difference in estimates of health inequity. Using the direct standardization, the magnitude of health inequity, expressed by the Gini coefficient, is 0.059 (95% confidence interval [CI]: 0.058, 0.061), while using the indirect standardization, the Gini coefficient is 0.086 (95% CI: 0.082, 0.091). The large difference between these inequity estimates reflects the large amount of unexplained variation in health – the adjusted R^2^ for the regression model is 0.258, which indicates that about 75% of the variation in the observed HUI is not explained by the model. The direct standardization method presumes this large unexplained variation is fair, while the indirect method regards this unexplained variation as unfair.

Both inequity estimates are policy relevant. The Gini coefficients of 0.059 (the direct standardization) and of 0.086 (the indirect standardization) translate into the expected mean differences in the HUI of 0.101 and 0.166, respectively, between two randomly selected persons. These HUI values are more than three times larger than the minimally policy relevant difference of the HUI, 0.030.

### Health inequality vs. health inequity

Figure 1 also shows that the choice of the standardization method influences the comparison between health inequality and health inequity. The Gini coefficient for the distribution of the observed HUI (0.094; 95% CI: 0.089, 0.100) is 1.6 times larger than the Gini coefficient for health inequity estimated by the direct standardization (0.059; 95% CI: 0.058, 0.061). However, the Gini coefficients for inequality and for inequity estimated by the indirect standardization (0.086; 95% CI: 0.082, 0.091) are not statistically significantly different. Therefore, the choice of the standardization method offers two contrasting results: About 60% of health inequality (the direct standardization) or almost all health inequality (the indirect standardization) we observe is inequitable.

## Discussion

In the context of the empirical assessments of health inequities, this paper investigated the empirical importance of the ethical question of whether unexplained health inequality is unfair. The classification of unexplained inequality as fair or unfair is closely connected to the choice of the fairness-standardization methods, a critical step for the measurement of health inequities. As the analysis of the US component of the JCUSH showed, this choice can substantially influence the empirical results regarding how much health inequity exists in the population and the proportion of observed health inequality that is inequitable. We obtained the same results in analyses using the Canadian sample of the JCUSH and using a different definition of health inequity, equal opportunity for health (results not shown).

The question of how best to treat unexplained health inequality deserves more extensive consideration in the assessment of health inequities than it currently does. Both direct and indirect fairness-standardization methods are technically valid but can produce different health inequity information and imply different ethical stances in regard to unexplained variation. An analogy here may be the choice between direct and indirect age-standardization methods in epidemiological studies [31]. Both of these methods are sound but are known to produce different results. Analysts are therefore advised to be explicit and consistent about their methodological choice. What complicates the choice of the fairness-standardization methods is that it is not merely methodological but ethical.

Although unexplained health inequality is not an issue for those who subscribe to the view that all health inequalities are inequitable (for whom all observed variation – explained or unexplained – is unfair), it is an unavoidable issue for empirical analysts who do distinguish between pure health inequality and health inequity. Currently available data and modeling techniques enable analysts to explain only a relatively small portion of observed variation in health at the individual level. Because the issue of unexplained inequality only arises in empirical work, it has rarely been paid attention to in the conceptual discussion regarding definitions of health inequity. Still, some work in the recent detailed philosophical analysis of health inequity by philosophers, economists, and ethicists provides a hint as to how to consider the ethical significance of unexplained inequality.

To examine the ethical significance of unexplained inequality, it is useful to recognize that unexplained variation – residuals in a regression context – consists of two types of variation: variation systematically related to unobserved factors and random variation. The issue of unmeasured systematic variation stems from methodological limitations. Improved data, such as longitudinal data with a rich array of variables capturing individuals’ life history, and improved modeling techniques can reduce unmeasured systematic variation. As soon as unmeasured systematic variation becomes observed systematic variation, the question goes back to a familiar, on-going debate regarding definitions of health inequity, that is, which sources of health inequality are ethically unacceptable.

To assess the ethical significance of random variation, the philosophical literature distinguishes “brute luck” – unfortunate events from which even sensible persons suffer, such as being hit by lightning during the commute with no warning, or suffering from a genetic disease by chance (often referred to as genetic lottery) – and “option luck” – unfortunate events associated with voluntary risks, such as being hit by lightning while playing golf with a plenty of warning or getting injured during voluntary bungee jumping [32-34]. The philosophical literature offers a wide range of views regarding the ethical significance of brute and option luck. Some scholars consider neither option nor brute luck as unfair because only variations in health associated with known socially distributed determinants of health are unfair [35,36]. Alternatively, most equality in opportunity theories, also known as luck egalitarianism, consider that inequality caused by brute luck is unfair while that by option luck is fair [37]. Yet another view sees both brute and option luck as unfair [38]. To date, this philosophical literature has not caught attention in health services and population health research and policy, but it is an important literature in the face of large unexplained health inequality in empirical work.

Advances in data, modeling techniques, and philosophical arguments are ongoing processes, and the measurement and monitoring of health inequities for effective policy making cannot wait for their perfection. Three proposals are available for the treatment of unexplained health inequality in the current imperfect world that still urges policy making. First, Bago d’Uva, Jones, and van Doorslaer [39] recommend in the context of need-standardization for health care utilization, which faces a directly analogous problem, that analysts always provide two estimates of inequity, the lower bound estimate provided by the direct standardization and the upper bound estimate by the indirect standardization. This is a pragmatic stop-gap solution but passes the difficult ethical question to users of health inequity information. Second, given complex causal relationships between health and its determinants and the fact that we do not understand them fully, we might argue that it would be safer to assume unexplained health inequality is of ethical significance, that is, unfair [40,41]. This judgment, and policy decisions that follow from it, will come with some opportunity cost. Resources that are devoted to address health inequity based on this judgment could be directed to competing health or other social issues. We should at least know the nature of such opportunity cost before committing to such judgment.

Finally, Garcia-Gomez and colleagues [7] empirically investigate what unexplained health inequality is. They tested the view articulated by Lefranc and colleagues in the analysis of unexplained income inequality [42]: classify unexplained inequality as luck; examine whether the distribution of luck is uncorrelated with ethically unacceptable sources of inequality; and if that is the case, consider luck an ethically acceptable source of inequality. In their analysis of inequality in mortality among the Dutch population, they adopted the view of equal opportunity for health as the definition of health inequity, which argues that health inequality due to factors beyond individual control is unfair. They considered variables such as sex, age, and education as ethically unacceptable sources of inequality while variables such as smoking, exercise, and weight as ethically acceptable sources of inequality. They found that unexplained inequality is distributed differently across groups of people categorized by sex, age, and education with or without controlling for the health behaviour. In sum, their analysis suggests that unexplained inequality is not an ethically acceptable source of inequality.

Most of this emerging empirical work and its authors’ insight in into the importance of ethical discussion are of considerable significance for public health and health policy. Given potentially serious policy implications of the issue of unexplained health inequality, analysts should at least make their methodological choices explicit and report both results from both standardization methods whenever they can. Moving beyond this pragmatic solution, however, analysts need to spur more debate and analysis regarding which treatment of the unexplained inequality has the stronger foundation in equity considerations.

## Endnote

^a^The choice of the standardization methods would become even more ethically relevant if we used a non-linear model for the HUI. This means that, in a sense, our results using a linear model provide conservative estimates of the importance of this choice. We would like to thank an anonymous reviewer for pointing this out.

## Additional file

Additional file 1:
**Categories at which variables are held constant in the fairness-standardization in the analysis.**

## Abbreviations

BMI: Body mass index
CI: Confidence intervals
CLAD: Censored least absolute deviation
HUI: Health utilities index
JCUSH: Joint Canada/United States Survey of Health
OLS: Ordinary least squares
US: United States
