# Supplementary material for: Unexplained health inequality – is it unfair?
Source: Int J Equity Health. 2015 Jan 31;14:11. doi: 10.1186/s12939-015-0138-2 (PMC4318200; doi:10.1186/s12939-015-0138-2)
Supplement: Additional file 1: — Categories at which variables are held constant in the fairness-standardization in the analysis. [file 12939_2015_138_MOESM1_ESM.docx]

**Additional file 1. Categories at which variables are held constant in the fairness-standardization in the analysis**

Footnotes for Appendix 1

To standardize fairness based on the definition of policy amenability, we hold each legitimate variable (for the direct standardization) and illegitimate variable (for the indirect standardization) constant at the category to which policies might reasonably aim.
